# Elimination of cyanobacteria and microcystins in irrigation water—effects of hydrogen peroxide treatment

**DOI:** 10.1007/s11356-019-07476-x

**Published:** 2020-01-06

**Authors:** Lisa Spoof, Sauli Jaakkola, Tamara Važić, Kerstin Häggqvist, Terhi Kirkkala, Anne-Mari Ventelä, Teija Kirkkala, Zorica Svirčev, Jussi Meriluoto

**Affiliations:** 1 Åbo Akademi University, Faculty of Science and Engineering, Biochemistry, Tykistökatu 6A, 20520 Turku, Finland; 2 Pyhäjärvi Institute, Sepäntie 7, 27500 Kauttua, Finland; 3 Faculty of Sciences, Department of Biology and Ecology, University of Novi Sad, Trg Dositeja Obradovića 2, Novi Sad, 21000 Serbia; 4 Department of Chemistry, University of Jyväskylä, P.O. Box 35, 40014 Jyväskylä, Finland

**Keywords:** Cyanobacteria, Microcystins, Irrigation water, Hydrogen peroxide, Spinach

## Abstract

Cyanobacterial blooms pose a risk to wild and domestic animals as well as humans due to the toxins they may produce. Humans may be subjected to cyanobacterial toxins through many routes, e.g., by consuming contaminated drinking water, fish, and crop plants or through recreational activities. In earlier studies, cyanobacterial cells have been shown to accumulate on leafy plants after spray irrigation with cyanobacteria-containing water, and microcystin (MC) has been detected in the plant root system after irrigation with MC-containing water. This paper reports a series of experiments where lysis of cyanobacteria in abstracted lake water was induced by the use of hydrogen peroxide and the fate of released MCs was followed. The hydrogen peroxide–treated water was then used for spray irrigation of cultivated spinach and possible toxin accumulation in the plants was monitored. The water abstracted from Lake Köyliönjärvi, SW Finland, contained fairly low concentrations of intracellular MC prior to the hydrogen peroxide treatment (0.04 μg L^−1^ in July to 2.4 μg L^−1^ in September 2014). Hydrogen peroxide at sufficient doses was able to lyse cyanobacteria efficiently but released MCs were still present even after the application of the highest hydrogen peroxide dose of 20 mg L^−1^. No traces of MC were detected in the spinach leaves. The viability of moving phytoplankton and zooplankton was also monitored after the application of hydrogen peroxide. Hydrogen peroxide at 10 mg L^−1^ or higher had a detrimental effect on the moving phytoplankton and zooplankton.

## Introduction

Cyanobacteria (blue-green algae) are photosynthetic prokaryotes. They are known producers of taste and odor compounds as well as toxins such as hepatotoxins and neurotoxins. Several cases of animal and human poisonings have been documented in the context of cyanobacterial proliferation (Roegner et al. 2013). Excess nitrogen and phosphorus originating, e.g., from wastewater or agricultural run-offs promote cyanobacterial growth and cyanobacterial blooms may form during favorable environmental conditions. The reduction of cyanobacteria and remediation of a lake through the reduction of the nutrient load is a long-term project and sometimes faster measures are needed, e.g., in the case of recreational waters or drinking water sources.

Microcystins (MCs) are cyclic heptapeptide hepatotoxins produced by some common cyanobacteria. Around 250 MC congeners differing mainly in amino acid constitution and the degree of methylation (Spoof & Catherine 2017) are known to date. MCs are tumor promoters (Ito et al. 1997) and MC-LR has been classified as a possible human carcinogen by the International Agency for Research on Cancer (IARC 2010). MCs have been suggested to be genotoxic, inducing damage to the DNA (reviewed by Žegura et al. 2011). Recently the possible neurotoxicity of MCs has been reviewed by Hu et al. (Hu et al. 2016). Humans may be regularly exposed to sub-lethal doses of MCs in drinking water (He et al. 2016) or by consuming contaminated fish, crop plants and other foodstuffs. A provisional guideline value for MC-LR in drinking water has been adopted by the WHO at 1.0 μg L^−1^ (WHO 2017). A human tolerable daily intake limit of 0.04 μg MC-LR kg^−1^ body mass per day has also been devised (WHO 2017).

MCs are chemically stable compounds and resistant to chemical hydrolysis (Harada et al. 1996) and boiling (Metcalf and Codd 2000). Purified MCs are also fairly stable under irradiation by sunlight (Tsuji et al. 1994). MCs are not actively secreted to the surrounding water but are released during cell senescence or due to environmental stress, leading to cell lysis. In natural waters extracellular MCs decrease with time through dilution, adsorption to organic matter, bacterial degradation, and photolysis in the presence of photosensitizers (Gągała and Mankiewicz-Boczek, 2012, Schmidt et al. 2014). Drinking water abstracted from water sources infested with cyanobacteria can contain extracellular MCs after conventional drinking water treatment (Zamyadi et al. 2012; Bullerjahn et al. 2016).

Colonies of toxic *Microcystis aeruginosa* (which could not be washed away) were detected on lettuce leaves irrigated with cyanobacteria-infested water (Codd et al. 1999). Another possible mechanism of plant contamination is through root uptake as MCs can be taken up by some plants from soil into the root system and further transported to the stem, leaves, and shoots and even to the fruits and seeds (reviewed by Corbel et al. 2014). MCs exhibit adverse effects on plants as reviewed by Machado et al. (2017). We have earlier demonstrated the presence of MC in hydroponically grown mustard (Kurki-Helasmo and Meriluoto 1998) but also in the roots of broccoli grown in soil (Järvenpää et al. 2007). The bioaccumulation of MC-LR was recently demonstrated in lettuce irrigated by pouring contaminated water on the plants but a partial decontamination of the plants was observed after seven-day irrigation with pure water (Cordeiro-Araújo et al. 2016). Spinach has been studied for the long-term effects of cyanobacterial crude extracts under simulated field conditions (Pflugmacher et al. 2007). Spinach was watered through the soil with a diluted cyanobacterial extract containing MC-LR. Germination, growth, and morphology of the plants were affected and alterations in the antioxidative response parameters were detected.

As MCs and other cyanotoxins may harm human, animal, and plant health, there is a need to reduce the presence of cyanobacteria and cyanotoxins in certain waterbodies. Application of hydrogen peroxide has been suggested to be used to combat cyanobacteria and cyanotoxins in natural waters (Matthijs et al. 2012). Although hydrogen peroxide is a strong oxidizing agent in its initial action, it is decayed to harmless water and oxygen fairly rapidly (typically within a few hours in natural waters). In nature hydrogen peroxide occurs in small concentrations in all surface waters and is produced by the biota (Mostofa et al. 2013). It also occurs in rain, dew, clouds, snow, and air. The formation of H_2_O_2_ and reactive oxygen species (ROS) in surface waters is induced by UV irradiation and the presence of natural photosensitizers such as dissolved organic carbon and especially humic acid substances. The biological production of H_2_O_2_ by bacteria and algae may however dominate over photochemical production especially in particle-rich eutrophic waters (Cory et al. 2016).

The hydroxyl radicals formed from H_2_O_2_ attack the photosynthetic mechanism of cyanobacteria by preventing the photosynthetic electron transfer and oxygen evolution. The accumulated ROS can destroy pigment synthesis and the integrity of membrane structures resulting in cell death. Other phytoplankton (green algae and diatoms) are less sensitive to H_2_O_2_ (Drábková et al. 2007a; Barrington and Ghadouani 2008). In the prokaryotic cyanobacteria photosystem II is less protected than in eukaryotic chlorophyta, diatoms, and cryptophytes due to the lack of membranes around cell organelles (Drábková et al. 2007b). Further, less amounts of enzymes that eliminate reactive oxygen species are produced in cyanobacteria (for a review on oxidative stress in cyanobacteria see Latifi et al. 2009).

The effects of H_2_O_2_ on cyanobacterial cell integrity have been studied in several laboratory-scale experiments with cyanobacterial cultures using, e.g., *Oscillatoria rubescens* (Barroin and Feuillade 1986), *Raphidiopsis (*Kay et al. 1984), *Planktothrix agardhii* (Matthijs et al. 2012; Weenink et al. 2015), and *M. aeruginosa* (Zhou et al. 2013; Huo et al. 2015). Yang and coworkers studied the effect of H_2_O_2_ on *Anabaena* and *Cylindrospermopsis* concurrently with *Planktothrix* and *Microcystis* in a laboratory (Yang et al. 2018). In addition, the sensitivity of benthic cyanobacteria towards H_2_O_2_ in filtered natural waters has been tested (Chen et al. 2016). The viability of cyanobacterial cells has been analyzed by the so-called PAM (Pulse Amplitude Modulation) method which measures the relative chlorophyll fluorescence quantum yield and thus photosynthetic capacity, or by the use of flow cytometry in combination with fluorescent probes (Mikula et al. 2012; Fan et al. 2013) and pigment analysis (Randhawa et al. 2012). The results from laboratory experiments have been successfully extrapolated into larger-scale experiments in which cyanobacterial overgrowth has been combatted for instance in a wastewater pond (Barrington et al. 2013) and in a small lake (Matthijs et al. 2012). While it seems possible to target cyanobacteria fairly selectively by hydrogen peroxide, attention must be paid not to harm other organisms (e.g., beneficial algae, zooplankton, and higher organisms) in the treated water by introducing too high doses of hydrogen peroxide.

When cyanobacterial cells are lysed, the possible intracellular cyanotoxins will be released. The fate of released MCs after H_2_O_2_ treatment of natural cyanobacterial populations has been studied in a few investigations only (Matthijs et al. 2012; Barrington et al. 2013; Lürling et al. 2014). The degradation of released MCs is of high interest for the preparation of safe drinking water. Chemical oxidation processes such as chlorination, ozonation, and permanganate lead to the complete destruction of extracellular MCs when applied at doses high enough (Sharma et al. 2012). Advanced oxidation processes involving H_2_O_2_/UV and reductive metal ions were shown to be even more efficient in MC degradation due to the production of hydroxyl radicals (Sharma et al. 2012). The outcome of the use of chemical oxidants is, however, strongly dependent upon the natural matrix of water making the extrapolation from one water source to another almost impossible (Sharma et al. 2012).

The aims of the current investigation were (a) to explore the potential of hydrogen peroxide in the elimination of cyanobacteria and MCs in abstracted lake water, (b) to study the possible accumulation of residual MCs in spinach irrigated with the treated water, and (c) to assess the safety of the hydrogen peroxide treatment for moving phytoplankton and zooplankton. The primary hypothesis to be tested was the following: if lake water containing cyanobacteria and MCs is treated with hydrogen peroxide, then the MC-producing cyanobacteria will be lysed and MCs eliminated in the treated water making the water suitable for irrigation purposes.

## Materials and methods

### Study location

Lake Köyliönjärvi (61° 05′–61° 10′ N, 22° 18′–22° 24′ E, SW Finland) is a shallow lake with a mean depth of 3 m and a maximum depth of 12.8 m. The area of the lake is 12.5 km^2^ and the surface area of the catchment 123.7 km^2^. Lake Köyliönjärvi is surrounded by intensively farmed fields and a total of 26 ditches run into the lake. The lake is rich in nutrients (Sarvala et al. 2000) and has recurrent mass occurrences of cyanobacteria from late June to autumn which have impaired the recreational use of the lake. There is also a potential of crop plant contamination if the cyanobacteria-rich water is used for irrigation purposes. The field experiments were using water abstracted from one location in Lake Köyliönjärvi (close to the jetty of the Räpi experimental farm) and performed at the Räpi experimental farm located adjacent to the lake.

### Hydrogen peroxide treatment of water from Lake Köyliönjärvi

**Fig. 1:**
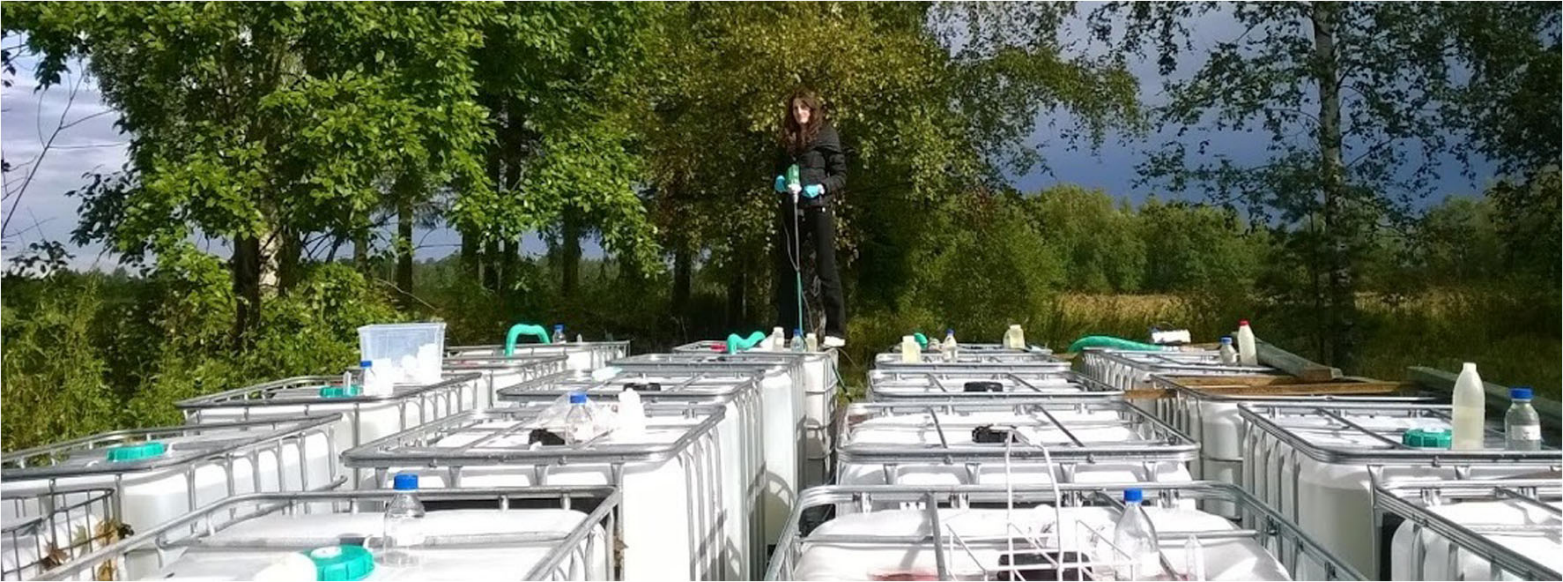
Photo showing the experimental setup. Twenty 1000-L tanks containing lake water and 0–20 mg hydrogen peroxide L^-1^ were incubated in ambient conditions. Hydrogen peroxide was being dosed to tank number 15 at the moment of the photography

Twenty white plastic agricultural tanks of 1000-L capacity were filled with water from Lake Köyliönjärvi by an electric agricultural pump and then dosed with 0–20 mg hydrogen peroxide L^−1^. A photo of the actual setup is shown in Fig. 1. Four series of experiments were performed during summer 2014.

Experiment 1: water abstraction and addition of H_2_O_2_ (start) on 17 June 2014, sampling on 01 July 2014, water subsequently used for irrigation

Experiment 2: start on 09 July 2014, sampling on 14 July 2014, irrigation

Experiment 3: start on 11 August 2014, sampling on 19–21 August 2014, irrigation

Experiment 4: start on 08 September 2014, sampling on 11–12 September 2014, irrigation

Experiments 1 and 2 provided the irrigation water for the first, early-summer spinach crop collected on 24 July 2014 while experiments 3 and 4 provided the water for the second, late-summer spinach crop collected on 16 September 2014.

The 35% H_2_O_2_ was purchased from Algol Chemicals (Espoo, Finland). Four doses of H_2_O_2_, 0 mg L^−1^, 2 mg L^−1^, 5 mg L^−1^, and 20 mg L^−1^, were used in the tanks. During each of experiments 1–4, five 1000-L tanks were prepared at each concentration level. The 35% H_2_O_2_ stock solution was first prediluted in 5 L of water and then added under continuous stirring for 2 min using an electric drilling machine equipped with a long shaft and an attached propeller.

### Irrigation of spinach with hydrogen peroxide–treated water

The first set of spinach plants was seeded on 06 June 2014. The irrigation with hydrogen peroxide–treated water was performed two times in July, on 07 July and 17 July. Untreated lake water and tap water were used as controls in the irrigation experiments. The late-summer spinach, seeded on 30 July 2014 was watered twice, on 29 August and 11 September. The irrigation was conducted by lifting up the water container on a tractor and by letting the water run in another container equipped with a pump forcing the water to the Avagro sprinkler system. The irrigation system was compiled in-house. The pumps were of model Saer FC25-2B 2.2 kW with 3–8 m^3^/h production. The first spinach crop was collected on 24 July 2014. The second spinach crop was harvested on 16 September 2014.

### Sampling protocols for determination of phytoplankton abundance, chlorophyll *a*, and MCs

The tank contents were mixed using the electric drilling machine before sampling. Three samples were withdrawn from each tank: 500 mL in a borosilicate glass bottle for MC analysis, 1 L in a plastic bottle for chlorophyll *a* determination and 50 mL control sample in a plastic Falcon tube for identification of dominant phytoplankton. At the start of the experiments samples were taken from at least three control tanks (0 mg L^−1^ H_2_O_2_). At the end of the experiment, close to the irrigation use of the water, samples were taken from three tanks per each H_2_O_2_ concentration (0, 2, 5, 20 mg L^−1^).

The samples for the analysis of the dominant phytoplankton consisted of 50 mL water acidified with 0.5 mL of Lugol’s iodine. Quantitative determination of the dominant phytoplankton was done according to the Utermöhl method using an inverted microscope (Leica DMI IRB, Wetzlar, Germany). Phytoplankton biovolumes were determined according to geometric shapes in Olenina et al. (2006), using average sizes instead of phytoplankton size classes.

For chlorophyll *a* analysis one hundred to 500 mL water samples were filtered using a mild vacuum onto 47 mm GF/C filters (Whatman, Maidstone, UK), dried for 2–3 h, frozen, and transported to laboratory. In the laboratory, 10 mL of ethanol containing MgCO_3_ (5 mg L^−1^) was added on top of the filters in 15 mL Falcon tubes, the tubes were vortexed and placed in a 75 °C water bath for 10 min. The tubes were then sonicated in an ultrasonic bath containing ice-cold water for 15 min. The samples were centrifuged for 10 min at 10 °C and 5000 rpm (CompactStar CS4, VWR, Leuven, Belgium) after which the absorbances at 665 nm and 750 nm were recorded with Biochrom Ultraspec 2100 pro UV/Vis Spectrophotometer (Biochrom Ltd., Cambridge, England). The analytical method used for chlorophyll *a* determination was an in-house modification of the method proposed by Sartory and Grobbelaar (1984).

The samples intended for the analysis of intracellular MCs consisted of 100–500 mL of tank water (depending on cell density) filtered onto 47 mm GF/C filters. The filters were dried at room temperature for 2–3 h and frozen for later analysis. The samples intended for the analysis of extracellular MCs were prepared in following way: 1–3 mL methanol was added to 100–300 mL filtrate (remaining from the samples intended for intracellular MC analysis), mixed to give a final methanol content of 1% and applied onto an activated Waters (Milford, MA, USA) OASIS HLB 200 mg cartridge. After washing the cartridge with 5 mL of water, the cartridge was dried for 2 min by sucking air through it, placed in a plastic bag, and frozen for later analysis.

### Extraction of cell-bound and dissolved MCs

Freeze-dried filters containing cyanobacterial material and the cell-bound fraction of MCs were extracted with 2.4 mL of 75% methanol for MCs (Spoof et al. 2003, Hautala et al. 2013). The extracts were sonicated for 15 min in a bath sonicator (Bandelin Sonorex RK 156, Berlin, Germany) and additionally for 1 min with a probe sonicator (Bandelin Sonopuls HD 2070 with a 3-mm microtip, 30% pulse, 30% energy). After centrifugation at 10,000*g* for 10 min, part of the supernatant was concentrated by evaporation with nitrogen gas at 50 °C. The residue was resuspended in 75% methanol (150 μL) and clarified by filtration through an Acrodisc GHP 0.45 μm syringe filter (Pall Life Sciences, Ann Arbor, MI, USA) prior to MC analysis by liquid chromatography-tandem mass spectrometry (LC-MS/MS).

The OASIS HLB SPE cartridges prepared at the site of sample collection and intended to capture to the extracellular MCs were eluted with 3 mL of 100% methanol. The methanol was evaporated with nitrogen gas at 50 °C. The dry residue was resuspended in 75% methanol (150 μL) and clarified by filtration through an Acrodisc GHP 0.45-μm syringe filter prior to LC-MS/MS.

### MC analysis in cyanobacterial cells and water by LC-MS/MS

The LC-MS/MS experiments were based on work by Spoof et al. (2003) and Hautala et al. (2013). The analyses were carried out on an Agilent Technologies (Waldbronn, Germany) 1200 Rapid Resolution (RR) LC coupled to a Bruker Daltonics HCT Ultra Iontrap MS (Bremen, Germany) with an electrospray (ESI) source. The LC system included binary pump, vacuum degasser, SL autosampler, and thermostated column compartment set at 40 °C. The separation of the toxins was achieved on Supelco (Bellefonte, PA, USA) Ascentis C_18_ column, (50 mm × 3 mm I.D. with 3 μm particles) protected by a 4 × 2 mm C_8_ guard column. The mobile phase consisted of (A) 99% water - 1% acetonitrile - 0.1% formic acid and (B) acetonitrile - 0.1% formic acid. A linear gradient solvent program was employed: 0 min 25% B, 5 min 70% B, 6 min 70% B, 6.1 min 25% B; stop time 10 min; flow-rate 0.5 ml min^−1^. The injection volume was 5 μL. The ion trap MS was operated in the positive electrospray ion mode. Ion source parameters were set as follows: dry temperature 350 °C, nebulizer pressure 30 psi, dry gas flow 8.0 L min^−1^, capillary voltage 4.0 kV. An MS scan from *m/z* 500 to 1200 followed by an auto-MS/MS function was employed. The ICC target was set to 300,000 with a maximum accumulation time of 100 ms. Abundant MS/MS fragmentation was achieved by the SmartFrag setting. The reference samples for the identification and quantitation of MCs consisted of extracts of *Microcystis* NIES-107 (deposited at National Institute of Environmental Studies, Tsukuba, Japan) and *Microcystis* PCC7820 (Institut Pasteur, Paris, France) analyzed in dilutions (Spoof et al. 2003, Hautala et al. 2013). The lowest quantifiable cell-bound MC concentrations in the lake/tank water were MC-dmRR 0.020 μg L^−1^, MC-RR 0.052 μg L^−1^, MC-dmLR 0.0051 μg L^−1^, MC-YR 0.0073 μg L^−1^, and MC-LR 0.028 μg L^−1^. The lowest quantifiable extracellular MC concentrations in the lake/tank water were MC-dmRR 0.012 μg L^−1^, MC-RR 0.033 μg L^−1^, MC-dmLR 0.0032 μg L^−1^, MC-YR 0.0046 μg L^−1^, and MC-LR 0.018 μg L^−1^.

### Analysis of selected MCs and spiked nodularin in spinach leaves by LC-MS/MS-MRM

A total of forty spinach plants were collected from different plot locations at two different dates and frozen. Freeze-thawed spinach was cut to pieces with a sharp knife and duplicate samples were taken for extraction. One-gram (fresh weight) samples were extracted using 5 mL of 100% methanol spiked with either 20 ng or 50 ng nodularin (NOD, internal standard, in-house purified) in 20-mL borosilicate glass vials. The vials were shaken overnight at room temperature in a WR Certomat shaker set at 180 rpm (B. Braun Biotech, Germany). Three-milliliter samples of the extracts were centrifuged the next day for 10 min at 12000*g* and room temperature. A total of 2.5 mL of the resulting supernatant was diluted to 20% methanol by adding 10 mL of MQ-water and bound onto an activated 100 mg Supelclean LC-18 cartridge (Supelco, Bellefonte, PA, USA). After application of the sample the cartridge was washed with 10% methanol and water, and air-dried for 1 min. The toxins were eluted with 2 × 0.5 ml of 75% methanol:24.8% water:0.2% trifluoroacetic acid with positive pressure. The eluate was evaporated with nitrogen gas at 50 °C. The dry residue was resuspended in 60% methanol (150 μL) and clarified by filtration through an Acrodisc GHP 0.45-μm syringe filter prior to LC-MS/MS-MRM (MRM, multiple reaction monitoring). Some spinach samples in duplicate were additionally spiked with an extract of *Microcystis aeruginosa* NIES-107 containing MC-RR and -LR (and other MCs) at previously determined concentrations. The samples were fortified prior to the LC-MS/MS analysis in order to check for matrix effects in the analytical method. The MRM analysis was performed on two main MCs (-LR and -RR) and nodularin only in order to increase the analytical sensitivity. The monitored MRM transitions were the following: MC-LR *m/z* 995.6 > 599.4, MC-RR *m/z* 519.8 > 440.2, and for nodularin *m/z* 825.4 > 389.2. The quantitation limits for MC-LR and MC-RR in the spinach extract matrix corresponded to below 0.02 μg g^−1^ spinach fresh weight.

### Viability index for moving plankton

As there is a risk that the hydrogen peroxide treatment damages non-target organisms such as zooplankton, the viability of moving plankton present in Lake Köyliönjärvi water was monitored at different hydrogen peroxide exposures. A viability index for moving plankton was constructed by assessing the abundance and motility of zooplankton (Protozoa, Rotatoria, Cladocera, and Copepoda) and moving phytoplankton under a microscope at a magnification of 400 times. Several tens of microscope view-fields were examined for each sample. Identification was done at the level of species or genus. The viability of each moving plankton species or genus was evaluated by an experienced hydrobiologist on a scale from 3 to − 1 using the following criteria (value/abundance/motility): 3/high/not affected; 2/intermediate/slightly affected; 1/low/barely alive; 0/not found/no information; − 1/present but destroyed or dead. The viability index is the sum of abundance/motility observations of all identified species/genera at a specific timepoint and hydrogen peroxide concentration. The viability index for moving plankton should be seen as a relative index intended for viability comparison within one experimental series.

## Results

### Phytoplankton

Various cyanobacteria were detected in Lake Köyliönjärvi. In preliminary studies in 2013, i.e., 1 year before this study, *Microcystis* spp. (*M. wesenbergii*, *M. botrys*, *and M. aeruginosa*) were identified as dominant species comprising > 75% of the cyanobacterial assemblage (sample taken on 22 August 2013)*. Planktolyngbya limnetica*, *Dolichospermum* sp., and *Aphanizomenon* sp. were also identified in the sample, in spring 2014 (13 May 2014). *Planktothrix* sp. and *Aulacoseira ambigua* were dominant in Lake Köyliönjärvi, and cryptomonads and *Cyclotella* spp. were conspicuous. During the experiments in 2014, cyanobacteria regularly dominated both abundance and biovolume in the abstracted water of Lake Köyliönjärvi (Table 1).

**Table 1:** Genera or species dominating abundance and/or biovolume in the controls of experiments 1–4

Experiment	Genera/species	% of total abundance	% of total biovolume
1 (June)	*Planktothrix* sp.	43	18
	*Dolichospermum* sp.	38	11
	*Dolichospermum mendotae*	11	7
	*Aulacoseira ambigua*	6	41
	*Pediastrum* spp.	0.3	12
2 (July)	*Planktolyngbya limnetica*	39	42
	*Aphanizomenon* sp.	33	36
	*Dolichospermum mendotae*	19	11
3 (August)	*Aphanothece* spp.	41	7
	*Planktolyngbya limnetica*	39	64
	*Pseudanabaena* sp.	19	27
4 (September)	*Aphanothece* spp.	69	71
	*Microcystis* spp.	13	5
	*Planktolyngbya limnetica*	9	2

### Temperature conditions

Surface water temperatures both in the lake (read from the jetty) and tank number 5 were monitored regularly at noon. The recorded temperatures varied between 16.3 and 21.2 °C in the lake and between 14.3 and 22.3 °C in the tank.

### Cell-bound chlorophyll *a* and MCs

Cell-bound chlorophyll *a* and MC values in the lake water, i.e., the water introduced to the tanks, were the following (first value chlorophyll *a*, second value total cell-bound MC): 38.8/0.97 μg L^−1^, 8.7/0.037 μg L^−1^, 46.9/1.27 μg L^−1^, and 29.8/2.4 μg L^−1^ in experiments 1, 2, 3, and 4, respectively. The high chlorophyll *a* values provided evidence of the eutrophic nature of Lake Köyliönjärvi. The ratios of MC concentration to chlorophyll *a* were also very low in the water introduced to the tanks, below 10% (0.0043–0.081) implicating a rather low abundance of MC-producing cyanobacteria in the phytoplankton.

**Fig. 2:**
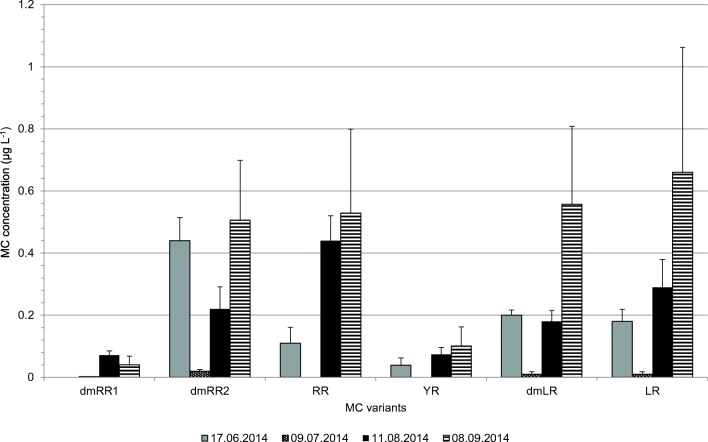
Detected MC variants in Lake Köyliö in summer 2014 in control water samples. The values represent a mean of three tanks (09 July, 11 August and 08 September) and five tanks (17 June). The error bars show standard deviation

The main variants detected in the phytoplankton were demethylated MC-RR (two different variants with different retention times), MC-RR, MC-YR, demethylated MC-LR, and MC-LR (Fig. 2). The MC profile in the lake water changed from the early summer to the autumn. In June and July demethylated MC-RR (the later-eluting variant, dmRR2) was the main MC-variant while in August MC-RR dominated. In September concentrations of MC-LR and MC-dmLR equaled or exceeded the concentrations of MC-RR variants.

**Fig. 3:**
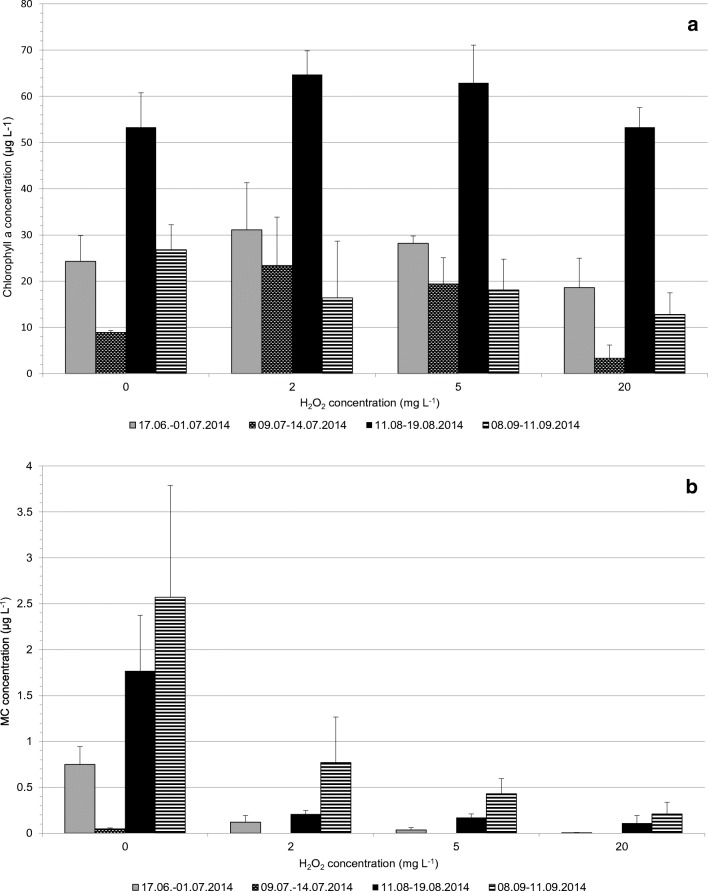
The effect of hydrogen peroxide on cell-bound chlorophyll *a* (**a**) and MC concentrations (**b**). The samples collected on the final day of the experiments. The values represent the mean of three tanks. The error bars show standard deviation

The cell-bound chlorophyll *a* and MC concentrations at the end of the experiments are shown in Fig. 3. The chlorophyll *a* values in some of the H_2_O_2_-treated tanks (H_2_O_2_ doses of 2–5 mg L^−1^), as compared with the control tanks, increased during the experiment (Fig. 3a). This may be indicative of an increased nutrient availability due to breakdown of some compounds/lysing of some organisms. Figure 3 b demonstrates the effect of hydrogen peroxide on the cell-bound MCs in four separate experiments made in June–July, lasting for 14 days, in July, lasting for 5 days, in August, lasting for 8 days and in September, lasting for 3 days. It can be seen that the use of H_2_O_2_, even at the lowest applied dose 2 mg L^−1^, effectively lysed a major part (70–88% in the different experiments) of the MC-producing cyanobacteria as monitored by residual cell-bound MC. The dose 5 mg L^−1^ lysed 80–96% of the MC-producing cyanobacteria. Nevertheless, some residual cell-bound MC was observed even at the highest applied dose 20 mg L^−1^, pointing to either an H_2_O_2_-resistant cyanobacterial subpopulation or a re-establishment of a population (possibly from akinetes).

### Extracellular MCs

**Fig. 4:**
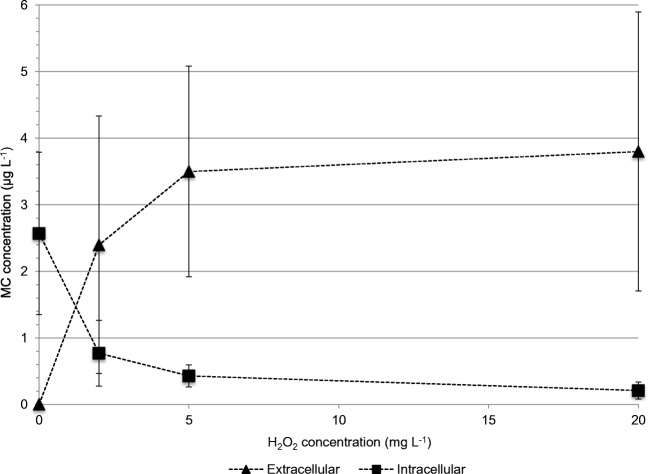
The effect of hydrogen peroxide on cell-bound and extracellular MC concentrations in the tank water. The data are from experiment 4 after 3 days treatment with H_2_O_2_. The values represent the mean of three tanks. The error bars show standard deviation

The untreated water from Lake Köyliönjärvi contained only very low concentrations, usually below 0.1 μg L^−1^, of extracellular MC (mean of three tanks). The highest extracellular MC, 0.12–0.28 μg L^−1^ were observed in August 2014. In most samples of the untreated water the concentrations of extracellular MC were close to the detection limit of the analytical method, ranging from not detectable to trace levels of MCs (data not shown). A comparison of cell-bound and extracellular MC in Experiment 4 shows that the increase in extracellular MC correlates with the decrease in cell-bound MC (Fig. 4). It is also possible from the results that H_2_O_2_ is releasing some additional MC from cell-bound material as compared to the traditional and widely used extraction protocol involving freeze-drying and sonication-aided extraction in aqueous methanol.

### Analysis of MCs in spinach leaves

Spinach plants were collected from various locations on the plot. Visual observation of the plants revealed no effects typically associated with MC damage such as growth inhibition and leaf necrosis (Pflugmacher et al. 2007).

Spinach samples were fortified with the internal standard NOD at levels 20 or 50 ng NOD g^−1^ spinach (fresh weight) in the beginning of the sample treatment procedure in order to estimate the recovery during processing. The spiked NOD with mass transition *m/z* 825.4 > 389.3 was detected in all the spiked samples. The recovery of the internal standard NOD was 48.2 ± 4.8% (mean ± SD, *n* = 40) in the summer spinach collected on 24.07.2014 and 56.0 ± 5.3% (mean ± SD, *n* = 40) in the autumn spinach collected on 16.09.2014. The recovery of MC-RR added prior to LC-MS/MS was 47.6 ± 6.5% (mean ± SD, *n* = 24) and the recovery of MC-LR 47.2. ± 14.5% (mean ± SD, *n* = 24) in spinach irrigated with water treated with 20 mg L^−1^ H_2_O_2_. The spinach plants used for the MC recovery studies were harvested on 24.7.2014.

**Fig. 5:**
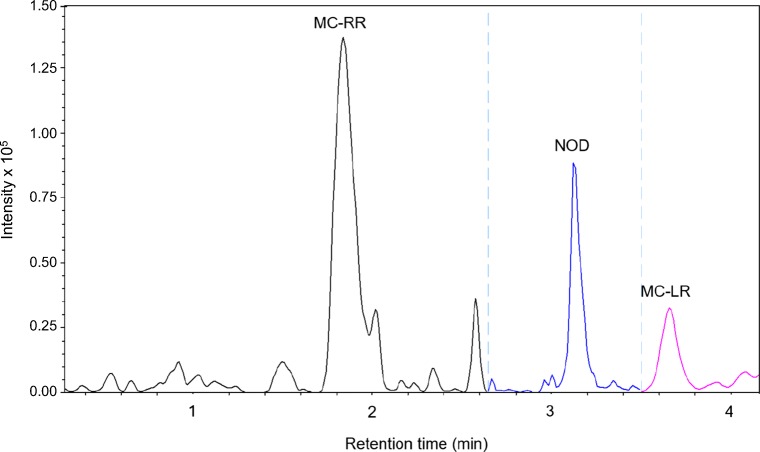
LC-MS/MS-MRM chromatogram of a spinach sample (with no detectable MC) spiked with NOD and an extract of NIES-107 containing MC-RR, MC-LR, and other MCs. The MRM signals corresponded to 0.45 ng MC-RR, 0.37 ng NOD, and 0.21 ng MC-LR on column

The LC-MS/MS-MRM technique provided a sensitive mode of analysis but on the used ion trap MS instrument the MRM technique can be applied on a single transition only in a specified time segment. Therefore MC-LR and MC-RR, which were abundant in the plankton samples, were used as indicator compounds for possible MC contamination in the spinach plants. An example of an LC-MS/MS trace of MC/NOD-spiked spinach extract is presented in Fig. 5. A total of 40 spinach plant samples in duplicates were prepared for analysis by LC-MS/MS. No MC-RR or MC-LR were detected in any of the spinach samples by LC-MS-MRM using mass transitions 519.8 > 404.3 for MC-RR and 995.6 > 599.4 for MC-LR.

### Viability index for moving plankton

**Fig. 6:**
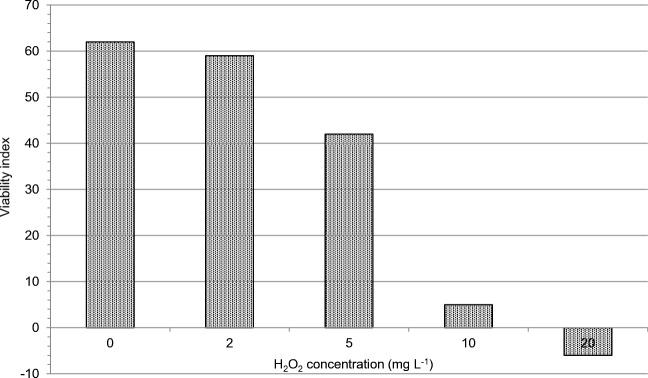
Viability index for moving plankton in a Lake Köyliönjärvi sample taken on 11 August 2014. The observations were made 2 h after application of H_2_O_2_ at concentrations of 0, 2, 5, 10, and 20 mg L^-1^

The viability of moving plankton (Protozoa, Rotatoria, Cladocera, Copepoda, and moving phytoplankton) decreased clearly at the hydrogen peroxide dose of 5 mg L^−1^ and more dramatically at doses 10 and 20 mg L^−1^ (Fig. 6). Both zooplankton (Protozoa, Rotatoria, Cladocera, and Copepoda) and moving phytoplankton were seriously affected at 10 and 20 mg L^−1^ (data not shown).

## Discussion

### Lake Köyliönjärvi and MCs

The ecological and chemical state of Lake Köyliönjärvi has been regarded as poor since the 1980s. A more detailed monitoring of the lake was initiated in the 1990s. High phosphorus concentrations, up to 170 mg m^−3^ total phosphorus especially in late summer, chlorophyll *a* values up to 180 mg m^−3^ and a low Secchi depth, 0.3–0.5 m, have been recorded (Sarvala et al. 2000). There has been only marginal improvement in the condition of the lake during the last few years and extensive cyanobacterial blooms have been observed annually. The lake is surrounded by farmed lands which are partly responsible for the eutrophication of the lake through an increased nutrient load. It would save irrigation costs if the farms could use lake water (instead of currently used networked water supply) for crop irrigation. A requirement for irrigation water is that the water is safe for food production and there is no risk for contamination of the crop plants by, e.g., cyanobacterial toxins or taste and odor compounds.

In 2013 we performed some preliminary testing of the water of Lake Köyliönjärvi for MC. Three different locations, Yttilän Otta, Kankaanpää, and Pappilanranta, on the shores of the lake were visited from June to October 2013. The highest observed cell-bound MC concentrations were 3.6 μg L^−1^ (19.08.2013) and 6.2 μg L^−1^ (26.09.2013). For comparison, cell-bound MC concentrations as high as 42 μgL^−1^ have been recorded on Åland Islands, Finland (Spoof et al. 2003). In 2014, the observed cell-bound MC concentrations in Lake Köyliönjärvi were lower than in 2013 (2.4 μg L^−1^ at maximum). The ratios of MC to chlorophyll *a* were low, below 10%, indicating either a low abundance of MC-producing cyanobacteria among the phytoplankton or cyanobacteria with fairly low MC production. As the cyanobacterial dominance was clear in most counted plankton samples, the latter alternative is more relevant in the case of Lake Köyliönjärvi. In our experience and as seen in the literature (Lindholm and Meriluoto 1991; Fastner et al. 1999), the concentration of MCs is typically 10–100% of the detected chlorophyll *a* concentration in cyanobacteria-dominated phytoplankton samples. The detected arginine-containing MCs MC-LR, MC-RR, and MC-YR, some with demethylation, occur frequently in the cyanobacterial MC pool in Finland (Spoof et al. 2003) and in Europe (Gkelis et al. 2005; Znachor et al. 2006). Out of all globally known MC variants, 74% have arginine or homoarginine in their structure (Spoof and Catherine 2017). The MC profile seemed to vary through the summer probably due to the different dominant cyanobacteria at the time of water abstraction, *Planktothrix* in the early summer and *Microcystis* in the autumn.

### Use of H_2_O_2_ for elimination of cyanobacteria and MCs

A systematic approach to the management of a particular lake necessitates a comprehensive analysis of the nutrient load and an understanding of the lake and biota characteristics. Long-term intervention techniques which have been used to mitigate cyanobacterial blooms in lakes, reservoirs, and rivers include nutrient reduction, alteration of hydrophysical conditions, artificial mixing, shortening of retention times, and various treatments, e.g., with clay (Paerl et al. 2016; Pan et al. 2016; Stroom and Kardinaal 2016). In addition to long-term measures, there is sometimes a need to rapidly destruct a cyanobacterial mass occurrence which may threaten human or animal health. Traditionally, copper sulfate has been used as an algicide (Hrudey et al. 1999) but lately hydrogen peroxide (H_2_O_2_) has been advocated as a convenient means of selectively killing cyanobacteria while not affecting other organisms (Drábková et al. 2007a, Matthijs et al. 2012). Hydrogen peroxide has been shown even to promote the growth of other phytoplankton over toxic cyanobacteria (Yang et al. 2018).

One of the benefits of H_2_O_2_ is that it leaves no harmful residue in the water but decomposes finally to water and oxygen. The decomposition is slow in pure water in the dark but accelerated in natural conditions. At an irradiance of 500 μmol m^−2^ s^−1^ (still below the daylight maxima) and in the absence of cyanobacteria a half-life of about 6 h was reported for 2.5 mg L^−1^ H_2_O_2_ (Drábková et al. 2007a). In the presence of natural irradiance of sunlight (daylight irradiance 500–2000 μmol m^−2^ s^−1^ with a broad spectrum of wavelengths including UV) H_2_O_2_ degrades readily in water with the concomitant formation of hydroxyl radicals (Huo et al. 2015), leading to a strong oxidizing and algicidal effect (Kay et al. 1984; Drábková et al. 2007a,b; Fan et al. 2013). The effect of H_2_O_2_ in cyanobacterial elimination in natural conditions can be hampered by several factors. In the microcosm experiments of Papadimitrou et al. (2016), 4 mg L^−1^ H_2_O_2_ was completely degraded in 5 h in the unfiltered water from a lake rich in organic matter (Papadimitrou et al. 2016). The presence of inorganic and organic water constituents has been shown to inhibit the degradation of target pollutants through radical scavenging mechanism (Sharma et al. 2012). The effect of H_2_O_2_ can also be buffered against by extracellular polymeric substances (EPS) typical for cyanobacteria. These are composed mainly of polysaccharides and proteins protecting the cells from the adverse effects of various environmental stressors (Gao et al. 2015). Species with high EPS are supposed to be better protected from the effects of H_2_O_2_.

In the experiments detailed in this paper, hydrogen peroxide lysed the majority of cyanobacterial cells with all tested concentrations (2 mg L^−1^, 5 mg L^−1^, and 20 mg L^−1^) with subsequent release of MCs to the water. The observed lysis corresponds to work by others. A treatment of an entire lake infested mostly with *Planktothrix agardhii* with 2 mg H_2_O_2_ L^−1^ resulted in the collapse of cyanobacterial population as well as the destruction of MCs by 99% within a few days, and the cyanobacterial population remained suppressed for 7 weeks (Matthijs et al. 2012). Unlike *P. agardhii*, a laboratory culture of *M. aeruginosa* required H_2_O_2_ doses above 4 mg L^−1^ to be killed (Lürling et al. 2014). Even higher H_2_O_2_ concentrations were suggested for *M. aeruginosa* in natural conditions where the species preferably forms colonies surrounded by a protective mucilaginous sheath instead of unicellular appearance as in laboratory conditions. The treatment of heavy cyanobacteria blooms with (the necessary) high H_2_O_2_ concentrations is questionable since the required H_2_O_2_ concentrations may harm other non-target organisms. In mesocosm and laboratory experiments involving water infested by *P. agardhii* zooplankton seemed to be sensitive to H_2_O_2_ concentrations higher than 2.5 mg L^−1^ (Matthijs et al. 2012; Weenink et al. 2015). Different zooplankton species have showed different susceptibilities towards hydrogen peroxide (Reichwaldt et al. 2012). Other phytoplankton (green algae and diatoms) have been shown to be less sensitive to H_2_O_2_ than cyanobacteria (Drábková et al. 2007a; Barrington and Ghadouani 2008).

The balance between efficient cyanobacterial elimination and minimal harm to other biota has been explored, and the optimal H_2_O_2_ dose was defined as the one that maintains an active concentration of 2 mg H_2_O_2_ L^−1^ for 5 h after a treatment (Weenink et al. 2015). While high enough concentrations of H_2_O_2_ efficiently kill cyanobacteria, sub-lethal concentrations of H_2_O_2_ may rapidly initiate antioxidant defenses in cyanobacteria. Such defense mechanisms may enhance at least the production of MC-LR (Giannuzzi et al. 2016). It has also been reported the binding of MC to proteins increases the fitness of *Microcystis* under oxidative stress (Zilliges et al. 2011). In order to avoid possible enhanced MC production or promotion of MC-producing strains it is essential that the active H_2_O_2_ concentration exceeds the cyanobacteriocidal threshold.

It was beyond the goals of the present study to unambiguously identify the cyanobacterial species responsible for MC production in Lake Köyliönjärvi. The cyanobacterial flora varied strongly during the summer (Table 1). A low dose of H_2_O_2_ (2 mg L^−1^) was more effective in killing MC-producing cyanobacteria in June as compared to September (as measured by residual cell-bound MC). *Planktothrix agardhii* (18% of total biovolume) was identified as the dominant cyanobacterial species in Lake Köyliönjärvi in June (Experiment 1), while *Aphanothece* spp. (71% of total biovolume), *Microcystis* spp. (5%) and *Planktolyngbya limnetica* (2%) were the main observed cyanobacteria in September (Experiment 4). The chlorophyll *a* values were comparable in June and September, 24.3 μg L^−1^ and 26.8 μg L^−1^, respectively. While high biomass and high dissolved organic matter consume more H_2_O_2_ also the cyanobacterial species present in the water and their morphology and physiology will have an effect on the success of the H_2_O_2_ treatment. *P. agardhii* has been shown to be more susceptible to H_2_O_2_ than *M. aeruginosa* (Lürling et al. 2014).

As intact cyanobacterial cells (in an exponential growth phase) do not actively secrete MCs into the surrounding water, the cell-bound MC concentrations are an indicator for viable cells not affected by H_2_O_2_ (Barrington and Ghadouani 2008; Qian et al. 2010; Zhou et al. 2013). Some natural release of toxins occurs due to normal cell aging and cell death. While the elimination of cyanobacteria themselves is important, it is even more important to eliminate the possible released MCs.

Released MCs were not efficiently degraded by H_2_O_2_ under the experimental conditions reported in this paper and this corresponds to some but not all earlier studies. Lürling et al. (2014) showed that 4 and 8 mg L^−1^ H_2_O_2_ effectively killed *M. aeruginosa* in culture (chlorophyll *a* concentration 702 μg L^−1^, initial total MC ca 1.1 mg L^−1^ of which 6% extracellular) with substantial release of MCs into the water. Over 75% of the released MCs remained in the water after 24 h. The authors stated that much higher H_2_O_2_ concentrations would be needed to eliminate extracellular MCs from the water placing other non-target organisms at risk. Fan et al. (2013) studied *M. aeruginosa* cultures with 36 μg L^−1^ cell-bound MC of and 10 μg L^−1^ extracellular MCs. The cultures treated with 10.2 mg L^−1^ hydrogen peroxide showed a decrease in cell-bound MCs to below detection limit in five days, but contrary to our results, Fan et al. (2013) found no concomitant increase in extracellular MCs. In mesocosm experiments the appearance of extracellular MCs after H_2_O_2_ application has been observed, followed by their reduction to below detection levels within a few days (Barrington et al. 2013; Matthijs et al. 2012).

The concentration of released MCs will be gradually lowered in naturals waters by several mechanisms: dilution, photochemical degradation by UV light, adsorption onto particles in suspension or in sediment, and especially in shallow lakes by biodegradation (Corbel et al. 2014). These processes are not immediate but can take from days to weeks depending upon physical, chemical, and biological conditions (Barrington et al. 2013). Microorganisms involved in MC degradation and their efficiencies were recently reviewed by Dziga et al. (2013). Some aquatic environments, especially those with no cyanobacterial history, probably lack strong populations of MC-degrading microorganisms. Even in a lake with a cyanobacterial history, such as Lake Köyliönjärvi, the degradation processes may not start immediately (see Fig. 5). As released MCs may be present in the hydrogen peroxide–treated water, the treated water should not be used for any critical purposes without testing of the MC concentration. The degradation of the released/residual MCs might be enhanced by the use of stronger oxidants such as ozone (Onstad et al. 2007) or bioreactors employing MC-degrading bacteria (Dziga et al. 2014). While both methods of assisted degradation are technologically feasible they may be considered too expensive for agricultural purposes. Contemporary methods for bioremediation also include the addition of pollutant-degrading enzymes into water (Sharma et al. 2018) and such an arrangement could possibly also involve the release of a MC-degrading enzyme, i.e., a microcystinase, into the water.

### Accumulation of MCs in spinach

Crop plants irrigated with cyanotoxin-infested water may accumulate these toxins either via the root system (Peuthert et al. 2007) as well as collect toxins on the surface of their leafy edible parts (Codd et al. 1999). Cyanotoxins have been demonstrated to induce a variety of toxic effects on plant metabolism affecting their development and growth (reviewed by Machado et al. 2017) which may lead to economical losses for the farmers. Plants can however show an increased tolerance for cyanotoxins (and even improved growth at certain MC concentrations) (Levizou et al. 2017; Cao et al. 2018). Vegetables and fruit contaminated with cyanobacterial hepatotoxins but showing no apparent external damage can present a risk for humans consuming such products.

Considerable MC accumulation has been demonstrated in lettuce irrigated with lake water containing a low concentration, 1.8 μg L^−1^, extracellular MC (Levizou et al. 2017). Lettuce was grown in peat-filled pots for 2 months and irrigated three times a week. The decontamination potential can vary between different plant species as demonstrated by Cordeiro-Araujo et al. (2016) who studied both the bioaccumulation and depuration kinetics of MC-LR in lettuce and arugula. MC-LR was shown to accumulate in lettuce grown in pots and irrigated by pouring, with water containing 5–10 μg L^−1^ MC-LR. Irrigation with uncontaminated water eliminated the toxin from lettuce.

Exposure of spinach to cyanobacterial crude extract has been shown to affect germination, growth, and morphology as well as antioxidative response parameters (Pflugmacher et al. 2007). The effects varied between different spinach variants, some of which even showed flowering. Spinach were grown in pots and were watered into the soil twice a week with 50 mL water containing cyanobacterial crude extract (0.5 μg L^−1^ MC-LR) for 6 weeks. Changes in morphology were visible after the third week of cultivation such as reduction in growth, leaf chlorosis and yellow leaves.

In the present study the MC concentrations in the irrigation water were fairly low, at highest ca 3.8 μg L^−1^ extracellular MC during experiment 4 in September. Such a MC concentration is quite typical for a dispersed cyanobacterial population. The spinach irrigated with the H_2_O_2_ treated water containing MCs did not show any signs of MC accumulation. No obvious phytotoxic effects were observed in the spinach plants or any obvious alternations in their growth.

### Ecological safety of the use of hydrogen peroxide

Hydrogen peroxide is a compound present in the water environment naturally through photochemical and biological activity, and added hydrogen peroxide is fairly rapidly degraded to water and oxygen. There is still reason for caution with the use of hydrogen peroxide as higher doses of it affect also other organisms than those targeted. In the present experiments hydrogen peroxide was detrimental to the viability of moving plankton at doses 5–20 mg L^−1^ and the scale of damage increased with the dose. No long-term ecological effects of hydrogen peroxide application were studied in this paper but such effects should be paid attention to as both zooplankton (Protozoa, Rotatoria, Cladocera, and Copepoda) and moving phytoplankton were affected by hydrogen peroxide at higher doses. Doses close to 2 mg L^−1^ seem safer at least from a short-term perspective.

## Conclusions

Lake Köyliönjärvi contained only relatively low concentrations of MCs in summer 2014. The concentrations were higher in the late summer than in the early summer. The primary hypothesis of the investigation was only partially verified: the treatment with H_2_O_2_ effectively lysed cyanobacterial cells causing MC to leak into the surrounding water but the H_2_O_2_ treatment did not destroy the released MCs. However, spinach irrigated with MC-containing water (either lake water or hydrogen peroxide–treated water) did not show any traces of the toxins, possible due to the relatively low concentration of MCs present in the lake/treated water. Viability of moving phytoplankton and zooplankton was severely affected by hydrogen peroxide at doses 10 and 20 mg L^−1^ and some detrimental effects were visible already at 5 mg L^−1^.
